# Management of Congenital Talipes Equino Varus (CTEV) by Ponseti Casting Technique in Neonates: Our Experience

**Published:** 2013-04-01

**Authors:** Md Saif Ullah, Kazi Md Noor-ul Ferdous, Md Shahjahan, Sk Abu Sayed

**Affiliations:** Department of Pediatric Surgery, Bangladesh Institute of Child Health (BICH) and Dhaka Shishu (Children) Hospital, Dhaka.

**Keywords:** Neonate, Talipes equino-varus, Ponseti technique

## Abstract

Objective: The purpose of this study is to evaluate the results of Ponseti technique in the management of congenital Talipes Equino Varus (CTEV) in neonatal age group.

Methods: It is a prospective observational study, conducted during the period of July 2010 to December 2011 at the Department of Pediatric Surgery in a tertiary hospital. All the neonates with CTEV were treated with Ponseti casting technique. Neonates with other congenital deformities, arthrogryposis and myelomeningocele were excluded.

Results: Total 58 CTEV feet of 38 neonates were treated. Twenty six were males and 12 were females. Thirty seven (63.8%) feet were of rigid variety and 21(36.2 %) feet were of non-rigid variety. Twenty patients had bilateral and 18 had unilateral involvement. Mean pre-treatment Pirani score of study group was 5.57. Mean number of plaster casts required per CTEV was 3.75 (range: 2-6). Thirty five rigid and 15 non-rigid (total 86.2%) feet required percutaneous tenotomy. Out of 58 feet 56 (96.6%) were managed successfully. Three (5.2%) patients developed complications like skin excoriation and blister formation. Mean post-treatment Pirani score of the study group was: 0.36 ± 0.43.

Conclusion: The Ponseti technique is an excellent, simple, effective, minimally invasive, and inexpensive procedure for the treatment CTEV deformity. Ideally it can be performed as a day case procedure without general anesthesia even in neonatal period.

## INTRODUCTION

The congenital talipes equinovarus (CTEV) or clubfoot is one of the most common and complex congenital deformities. The incidence of idiopathic clubfoot is estimated to be 1 to 2 per 1,000 live births. [1] The deformity has four components: ankle equinus, hindfoot varus, forefoot adductus, and midfoot cavus. [2] The goal of the treatment is to correct all the components of clubfoot to obtain painless, plantigrade, pliable and cosmetically and functionally acceptable foot within the minimum time duration with least interruption of the socioeconomical life of the parent and child. [2,3]

There is nearly universal agreement that the initial treatment of the clubfoot should be non-operative regardless of the severity of the deformity. If there is no improvement, then most of the surgeons prefer postero-medial release (PMR) of the soft tissue. The primary disadvantages of PMR are high complication and recurrence (13-50%) rate and the difficulty of treating recurrences. [4] Most of the authors have concluded that extensive surgery is not the right approach to the management of CTEV. [5] Over the past two decades, more and more success has been achieved in correcting CTEV without the need for surgery by Ponseti casting technique, which has become a gold standard worldwide. It includes serial corrective manipulation, a specific technique of the serial application of plaster cast supported by limited operative intervention (percutaneous Achilles tenotomy) The method has been reported to have success rate approaching 90- 96% in short, mid and long-term results. [5-10]

The Ponseti casting technique of club foot management has been shown to be effective, producing better results and fewer complications than traditional surgical methods. [11] In recent years, interest has been renewed in the Ponseti casting technique, and many centers now believe that most clubfeet can be treated by Ponseti casting technique rather than surgery [12]. Ponseti casting technique is especially important in developing countries, where operative facilities are not available in the remote areas. The physicians and personnel trained in this technique can manage the cases effectively with the cast treatment only. [13]

The purpose of this study was to evaluate the result of Ponseti casting technique used over last 2 years in our institute for the treatment of congenital clubfoot in neonates.

## MATERIALS AND METHODS

This is a prospective observational study, conducted in a tertiary hospital. The study period was from July 2010 to December 2011. All the neonates with CTEV presented to the Department of Pediatric Surgery were treated according to the Ponseti casting technique. Neonates with clubfeet associated with meningocele, meningomyelocele, arthrogryposis multiplex congenita and other neuromuscular causes were excluded. A prior approval was taken from the Institutional Review Board. An informed written consent was taken from all parents. All relevant data were collected from each participants using predesigned data sheet that included patient’s demography, physical examination, management, which included Pirani severity scoring score [11] (for initial assessment of the severity, and for evaluation of the feet after each component of the treatment and ultimate final outcome), total number of the casts applied before tenotomy, pre and post procedure complications like plaster sore, skin excoriation, blister formation, excessive bleeding following tenotomy or any other complication.

**Treatment protocol and follow up:**

We followed a protocol according to the Ponseti casting technique (Fig. 1-3).

The treatment included gentle manipulation of the foot and the serial application of above knee plaster casts at weekly interval without anesthesia, as described by Ponseti [2].

The foot was markedly abducted up to 70 degrees without pronation (combined movements of abduction, extension and eversion of the foot) in the last cast, which is very important for complete correction and it prevent early recurrence. If the varus deformity of the heel had been corrected and residual equinus was observed after the adduction of the foot and, a simple percutaneous Achilles tenotomy was performed under local anesthesia. After the tenotomy, an additional above knee cast with knee flexed in 90 degrees was applied and left in place for three weeks to allow for healing of the tendon. As the tenotomy wound was very minimal (less than 0.5cm), done percutaneously and was not stitched, so no window was made in the cast. After removal of the cast, a Denis-Browne bar and shoes (D-B splint) was used to prevent relapse of the deformity. This is best accomplished with the feet in well-fitted, open-toed, medial bar, high-top straight-last shoes attached to Denis-Browne bar. The D-B splint was worn full time (day and night) or at least 23 hours per day for the first 3 months and then for 12 hours at night and 2 to 4 hours at day for a total of 14 to 16 hours during each 24 hour period. The protocol continues until the child is 3 to 4 years of age. 

The patients were followed up on a weekly basis during the initial stages of treatment. After applying D-B splint, on a monthly basis for three months and then once every three months till the patients was three years of age. The parent advised to come for follow up every six months to one year till 5 years and then after 1-2 years till skeletal maturity is achieved. 

**Figure:**
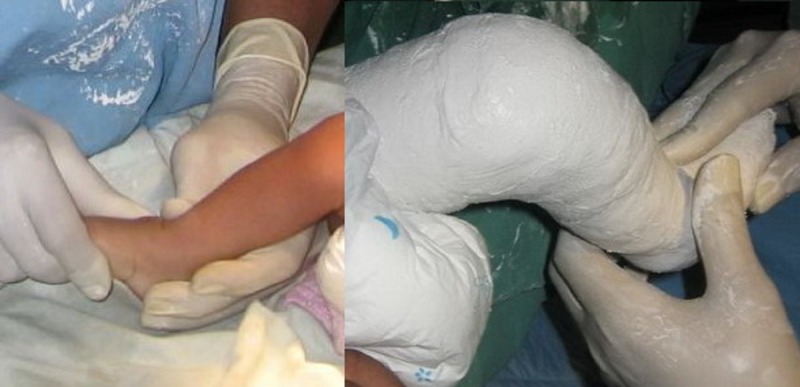
Figure 1: Manipulation and application of cast

**Figure:**
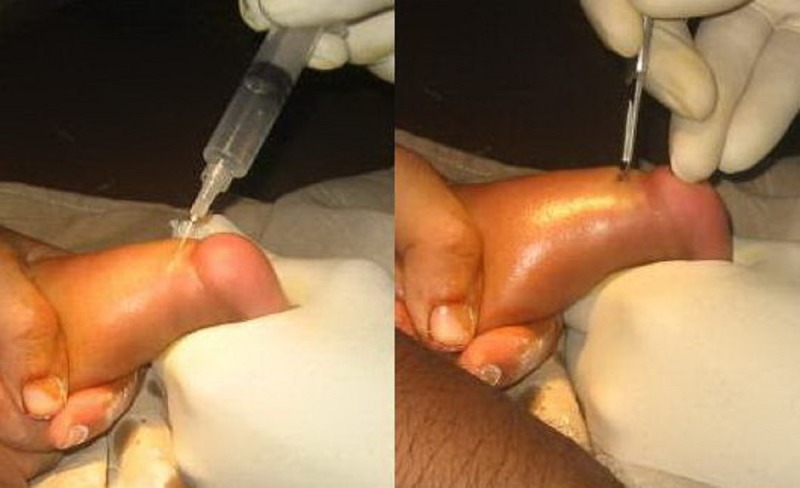
Figure 2: Steps of tenotomy

**Figure:**
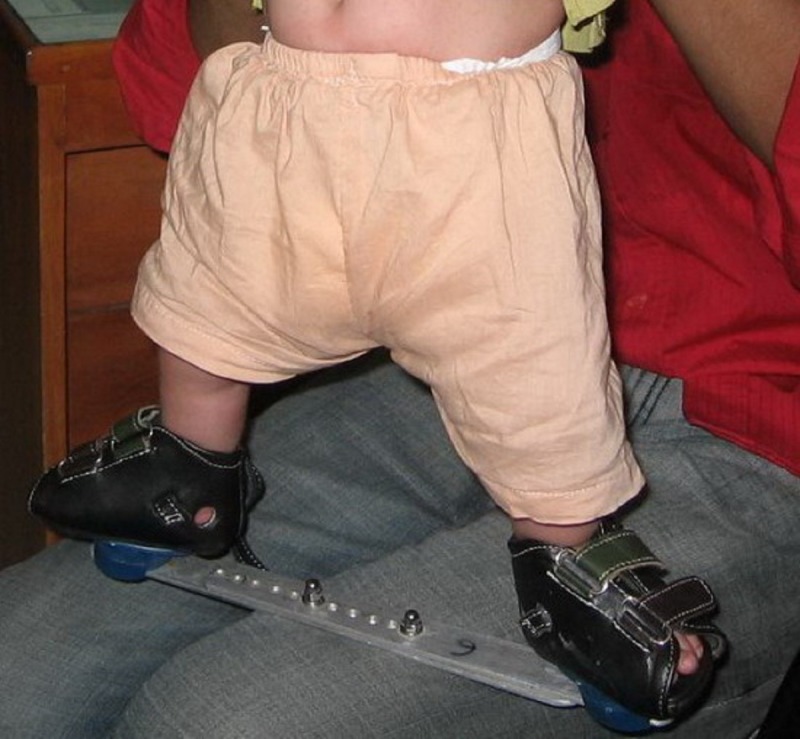
Figure 3: D-B splint

**Final outcome measurement:**

The outcome was measured by Pirani score [11]. This is the main variable of the study which can detect the degree of correction. It scores 6 clinical signs: 3 for midfoot, 3 for hindfoot. Three signs of midfoot score (MS) and hindfoot score (HS) grading the amount of deformity between 0 and 3. The Pirani score 0 means normal foot, the Pirani score 3 means moderately abnormal foot, the Pirani score 6 means severely abnormal foot. 

In our study the final outcome was categorized as excellent, good and poor. When Pirani score became 0, it was graded as excellent, when it became 0.5 to 1, it was graded as good and poor outcome occurs when the score became more than 1. Excellent and good outcomes obviously reflected to successful management. Poor outcome reflected treatment failure; these patients were advised further surgical management.

The collected data was analyzed and presented in tables.

## RESULTS

During the study period a total of 70 patients with 109 clubfeet were treated and followed up diligently. Of these, 38 neonates with 58 CTEV have been reported and analysed in this study. There were 28 boys and 12 girls with a male female ratio of approximately 2:1.

Of the 58 clubfeet, 37 were rigid and 21 of non-rigid variety. Of the 18 patients having only unilateral involvement, 11 had right sided affliction and 7 had their left feet involved. Mean pre-treatment Pirani score in the study group was 5.57 (SD ± 0.56). There was no significant difference between mean Pirani scores for the rigid and the non-rigid verities (5.69 ± 0.47 vs. 5.37 ± 0.69). (Table1). 

**Figure:**
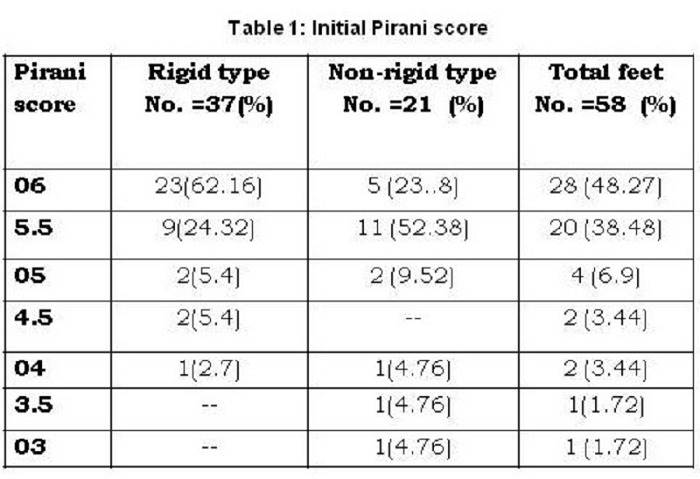
Table 1: Initial Pirani score

Mean number of plaster casts required per CTEV was 3.75 ± 0.80. More casts were required for the rigid feet as compared to non-rigid feet (5.11 ± 6.21 vs. 3.40 ± 0.77 (Fig. 4).

**Figure:**
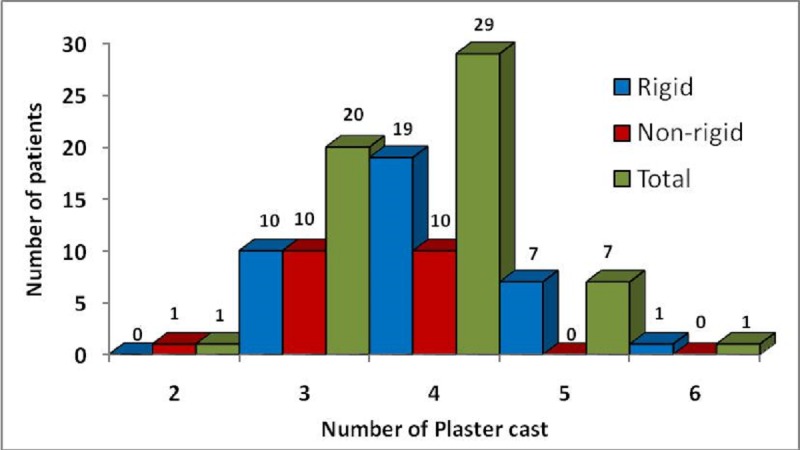
Figure 4: Number of plaster cast needed for correction.

A total of 50 (86.2%) feet (35 rigid and 15 non-rigid) required percutaneous tenotomy. Only 8 (13.79%) feet (2 rigid and 6 non-rigid) were improved by plaster cast alone. Out of 58 feet 56 (96.55%) were managed successfully (Table 2).

Only 3 (5.17%) patients developed complication. One (1.71%) developed skin excoriation and other 2 (3.4%) developed blister formation. 

The Pirani score after completion of overall treatment (with or without tenotomy) was recorded. Mean post-treatment Pirani score of the study group was 0.36 ± 0.43. As expected, the non-rigid feet fared better than the rigid feet, with their post-treatment scores of 0.17 ± 0.24 and 0.34 ± 0.45 respectively (Table 3). The average approximate total cost of treatment per patient was also estimated [Table 4]. Mean follow up period was 1year 11 months (range: 2years 4months to 10 months).

**Figure:**
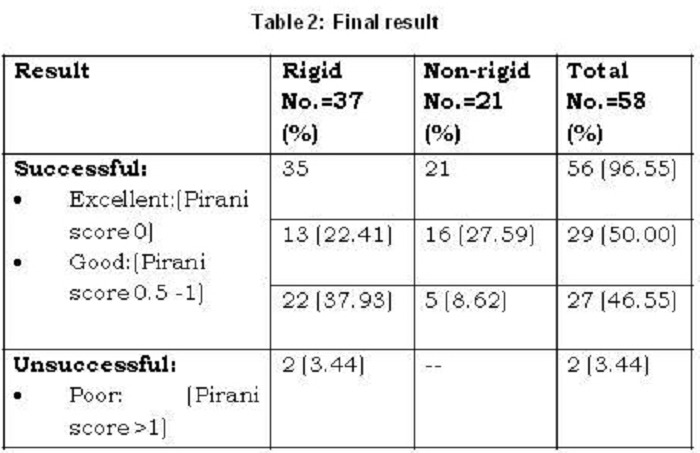
Table 2: Final result

**Figure:**
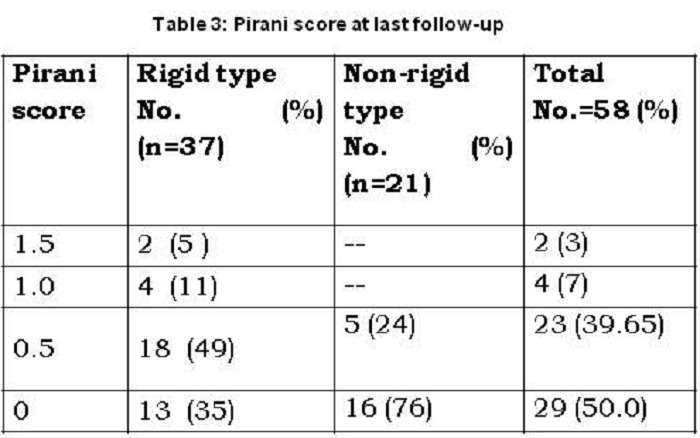
Table 3: Pirani score at last follow-up

**Figure:**
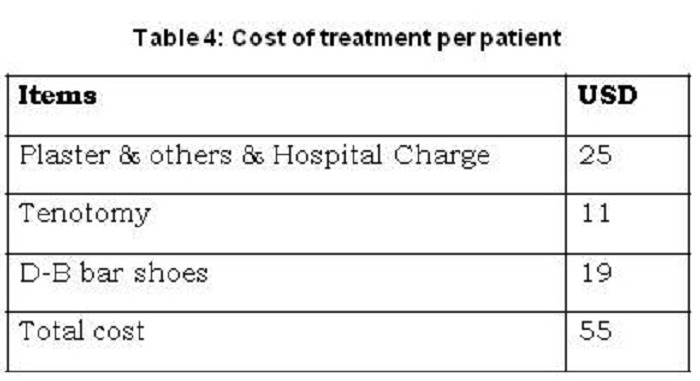
Table 4: Cost of treatment per patient

## DISCUSSION

CTEV is one of the commonest congenital deformities. It is a complex deformity comprises of equinus, varus, adductus and cavus, which are difficult to correct. It requires meticulous and dedicated effort on the part of treating physician and parents for the correction of the deformity [13]. The goal of treatment is to reduce or eliminate these deformities so that patient has a functional, pain free, plantigrade foot with good mobility without calluses and does not need to wear modified shoes [14].

The Ponseti casting technique of correction of CTEV deformity requires serial corrective casts with long term brace maintenance of the correction The treatment needs to be started as soon as possible and should be followed under close supervision [2,15]. The Ponseti casting technique yielded satisfactory anatomical and functional result with simple, effective, minimally invasive, inexpensive and ideally suited for all countries and cultures [2]. 

The available literature suggests that the results were better if this method of treatment was started as early as possible after birth [8, 13]. The factors responsible for clubfoot deformity are active from the 12th to 20th weeks of fetal life upto 3-5 years of age [16, 17]. 

More than half of the CTEV patients in our series presented in the neonatal age. This has been the experience of other authors also [13] and probably relates to the growing awareness of the entity in the parents nowadays. 

Mean pre-treatment Pirani score grouping this series were similar to those reported previously [7, 14, 18]. The mean number of plaster casts required per feet in our series was 3.75, much less as compared to the other series [13-15]; this is owing to the fact that we have analysed only neonates in the present study. All the available studies including ours have shown rigid feet required more casts than non-rigid feet to correct the deformity.

In our study, 86.2% feet (35 rigid and 15 non-rigid) required percutaneous tenotomy. Tenotomy was needed in 95% of Gupta’s patients [13] and 91% of Dobbs’s patients [19]. All the studies show that tenotomy was required in those patients who initially have severe deformity. Bor et al quoted, “A foot that requires many casts for the initial correction is more likely to require future additional surgery” [7]. As we included only neonates, and started treatment early, our patients needed tenotomy less frequently. A large number of pediatric orthopedic surgeons think that success of Ponseti casting technique depends on whether casting begins within hours of birth [20].

In our study, 96.6% CTEV feet were managed successfully (Table 2). The complication rate was low. Only one neonate who had rigid feet at presentation required posteromedial release (PMR) for both feet later. All the parents of the patients with successful repair were satisfied with the corrected feet of their children. The success rates for this technique in children have been quoted to range from 78% to 96.7% [5, 7, 9,10].

The most difficult part of the Ponseti casting technique is maintenance of bracing protocol [7]. The parents of our study group reported that initial two or three days were the critical period, during which patients were restless and tried to remove the splint. After that the patients were adjusted with splint. We agree with most of the authors that correction of the foot also depends on the brace protocol [6,7,13,14,17]. Parental compliance can be improved by educating the parents as to the proper use of bracing and the hazards of improper or insufficient bracing.

Another difficult part of the study was follow-up. Correction of foot by serial cast with or without tenotomy is only a part of the total management. With the initial correction of the foot, parents misunderstand that the main and difficult part of the treatment is over and hence they do not come for follow up. To overcome this problem, we motivated the parents and their family members. Though none of our patients dropped out from follow up, follow up in one of the patients was rather irregular; this very patient eventually required further surgical treatment. 

Similar to other’s experience [21], we found this treatment technique to be very cost-effective.

## CONCLUSION

It can be concluded that CTEV deformity can be effectively treated by Ponseti casting technique with excellent results and without significant morbidity. This method is simple, effective, minimally invasive, and inexpensive and ideally can be performed at outpatient department without general anaesthesia, even in neonatal period.

## Footnotes

**Source of Support:** Nil

**Conflict of Interest:** None

**Editorial Comment:** If pediatric surgery is a specialty of congenital malformations, it then defies logic as to why most of the pediatric surgeons all over the world do not treat clubfoot deformities. In fact, it was a pioneer British pediatric surgeon - Sir Denis Browne – who hypothesized that this malformation is due to abnormal position of fetus during gestation. As the logical extension of this, he was the first to demonstrate the superiority of non-surgical treatment in 1937. It is only a decade later Ponseti further developed the concept and described his method of manipulation therapy. The splint designed by Sir Denis Browne to treat clubfoot is still in use and is once again proved to be effective by Saif Ullah et al. We believe that this article will revive the interest of younger pediatric surgeons in the management of congenital clubfoot.
